# Effective Access to Care in a Crisis Period: Hypertension Control During the COVID-19 Pandemic by Telemedicine

**DOI:** 10.1016/j.mayocpiqo.2021.11.006

**Published:** 2021-11-15

**Authors:** Prentiss Taylor, Collin Berg, James Thompson, Kristin Dean, Tony Yuan, Shriram Nallamshetty, Ian Tong

**Affiliations:** a Doctor on Demand, San Francisco, CA; b Cardiology Section, Veterans Administration Palo Alto Healthcare System, Palo Alto, CA; c Division of Cardiovascular Medicine, Stanford Medicine, Stanford, CA; d Department of Medicine, Stanford Medicine, Stanford, CA

**Keywords:** ACC, American College of Cardiology, AHA, American Heart Association, AMA, American Medical Association, BP, blood pressure, DBP, diastolic blood pressure, SBP, systolic blood pressure

## Abstract

**Objective:**

To assess the effectiveness of telemedicine video visits in the management of hypertensive patients at home during the first year of the COVID-19 pandemic.

**Methods:**

A quantitative analysis was performed of all home video visits coded with a diagnosis of essential hypertension during the first 12 months of the COVID-19 pandemic (March 2020 through February 2021). A total of 10,634 patients with 16,194 hypertension visits were present in our national telemedicine practice database during this time. Among this population, a total of 569 patients who had 1785 hypertension visits met the criteria of having 2 or more blood pressure (BP) readings, with the last BP reading occurring in the report period. We analyzed baseline characteristics and BP trends of these 569 patients during the study period. Voluntarily submitted patient satisfaction ratings, which were systematically requested at the end of each visit, were also analyzed.

**Results:**

The mean age of the patients in this study cohort of 569 patients was 43.9 years, and 48.3% (275) were women. More than 62% (355) of the patients had an initial systolic BP (SBP) above 140 mm Hg, and 25.3% (144) had an initial SBP of greater than 160 mm Hg. The average number of visits during the study period was 3.1 visits per patient; an average of 6.4 BP measurements per patient were available. During the study period, 77% (438) of the patients experienced an improvement in either SBP or diastolic BP (DBP), with mean reductions of −9.7 mm Hg and −6.8 mm Hg in SBP and DBP, respectively. A total of 416 patients in the cohort started with a BP above 140/90 mm Hg. For this subset of patients, 55.7% (232) achieved a BP of 140/90 mm Hg or lower by the end of the study period, and the average reductions in SBP and DBP were −17.9 mm Hg and −12.8 mm Hg, respectively, which corresponded to improvements of 11.2% and 12.4%. These improvements did not vary significantly when patients were stratified by age, sex, or geographic region of residence (rural vs urban/suburban). Voluntarily submitted patient surveys indicated a high degree of patient satisfaction, with a mean satisfaction score of 4.94 (5-point scale).

**Conclusion:**

Clinician-patient relationships established in a video-first telemedicine model were broadly effective for addressing suboptimally controlled hypertension. Patient satisfaction with these visits was high.

According to the Centers for Disease Control and Prevention and the American Heart Association (AHA), hypertension remains a significant population health issue in the United States and globally.^1^ Researchers in 2020 reported that US population awareness of having hypertension and rates of adequate control of hypertension have declined during the past decade.^2^^,^^3^ The COVID-19 pandemic has exacerbated these issues by causing a greater than 25% decrease in primary care visits in the United States in 2020.^4^ Multiple reports document the disruption of chronic care visits during the pandemic^5^ and the potential downstream negative impacts on population-level health as well as on individual well-being. Many patients with chronic illnesses faced significant barriers to accessing care because of widespread closure of primary care physician offices during the initial months of the pandemic. Telemedicine visits were deployed by many health systems to address these issues. As our group has previously reported, video visits were able to fill those gaps in access to care during the pandemic.^6^ However, recent studies indicate that virtual visits, nationally, were much less focused on addressing hypertension and cardiovascular risk during 2020 than in recent nonpandemic periods.^7^

This report describes the experience of our national telemedicine practice with primary care virtualist physicians trained to provide holistic hypertension care. We queried the effectiveness of video visits for hypertension for a national, commercially insured population during the pandemic. In this study, our providers used the AHA-endorsed technique for home blood pressure (BP) monitoring to guide hypertension management by video visits with primary care physicians during the first COVID-19 pandemic year 2020-2021. We also assessed patient satisfaction with this video-first approach to management of hypertension.

## Methods

### Study Population

**Figure 1:**
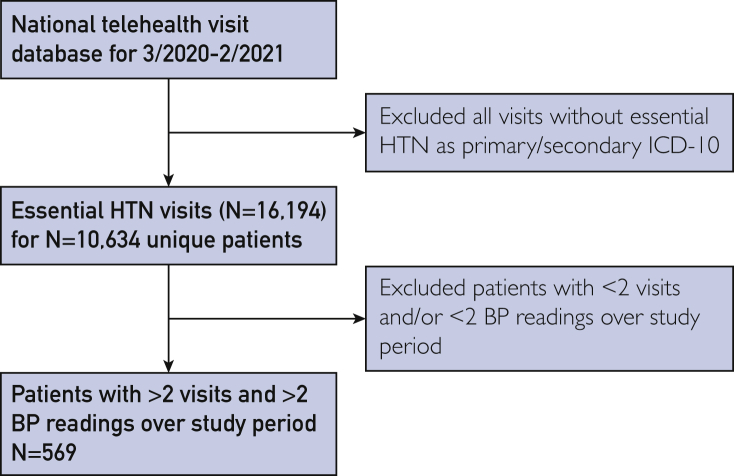
Study cohort flow diagram. Overview of study cohort selection from Doctor on Demand National Virtual Practice Database. BP, blood pressure; HTN, hypertension; ICD-10, *International Classification of Diseases, Tenth Revision*.

Encounters in the national Doctor on Demand Professionals database for the study period spanning from March 1, 2020, through February 28, 2021, were queried for essential hypertension visits (*International Classification of Diseases, Tenth Revision* code I10). We limited our analysis to patients with essential hypertension code I10 listed as the primary or secondary reason for visiting, with at least 2 hypertension-related visits and at least 2 BP readings, with the last reading occurring during the study period (Figure 1).

### Telemedicine Visits and Providers

Video visits were conducted on home mobile devices or personal computers with a board-certified primary care physician or a certified nurse practitioner. More than 95% of visits were performed by physicians. All clinicians followed American College of Cardiology (ACC)/AHA guidelines for hypertension treatment.^1^ Self-measurement of BP used American Medical Association (AMA)–approved devices and the technique endorsed in ACC/AHA guidelines for hypertension.^1^ The clinicians were trained to discuss AMA- or Consumer Reports–recommended, validated BP monitors with patients. Review of medications, family history, and comorbidities was captured in an electronic health record. Primary care physicians discussed diet (Dietary Approaches to Stop Hypertension diet), lifestyle, medication adherence, adverse effects, and shared decision-making with each patient. Screening for psychosocial stress was completed when indicated using the 2- and 9-item Patient Health Questionnaire and the 7-item Generalized Anxiety Disorder questionnaire. Patients automatically received hypertension-specific follow-up instructions for home study and patient satisfaction surveys after each visit. Patients were prompted to provide feedback immediately after visits through a secure cloud-based digital platform using a 5-star rating system.

### Study Outcomes

Reductions in systolic BP (SBP) and diastolic BP (DBP) across all patients were determined by comparing the initial BP with the last BP recorded within the study period (March 2020 through February 2021). Average SBP and DBP reductions were stratified by age (<40 years, 41 to 60 years, and >60 years), sex (female vs male), geographic region (metro/urban vs rural), and initial SBP (<120 mm Hg, 120 to 129 mm Hg, 130 to 140 mm Hg, 140 to 160 mm Hg, and >160 mm Hg). Patient zip codes were self-entered, and metro/urban vs rural classification was based on a Department of Labor Office of Workers’ Compensation Program zip code data set.

### Statistics

Paired *t*-tests were employed to analyze differences in mean SBP and DBP in patients at the start and end of the study period. Comparisons of average BP reductions stratified by age range, sex, geographic location, and initial SBP were performed using unpaired *t*-tests (sex, geographic location) and analysis of variance (age range, initial BP range).

## Results

In our study, we identified a total of 569 unique patients who had a total of 1785 video visits and 3626 BP readings during the 2020-2021 pandemic study period. The study population was representative of the general US population with regard to key demographic factors and clinical characteristics (Table). In the cohort, 48.3% of patients (275) identified as female and 51.7% (294) identified as male. The mean age of patients included in the analysis was 43.9±10.5 years. More than 86% of the video visits were conducted in metro/urban or suburban zip codes; approximately 14% of visits were in rural zip codes. The cohort consisted primarily of primary prevention patients (0.5% had a prior history of ischemic heart disease). The prevalence of concomitant risk factors was significant but lower than observed in the general US population. The most prevalent nonhypertension cardiovascular risk factor in the cohort was dyslipidemia, with more than 15% of patients carrying a diagnosis of a lipid disorder. Approximately 12% (68) of the patients in the cohort were either overweight or obese. In addition, 10.5% of the cohort (60 patients) had either prediabetes or diabetes. The patients in the cohort had an average of 3.1 visits during the first year of the pandemic (March 2020 through February 2021). The average number of BP measurements in the multiple visits group was 6.4. The average duration of visits was 12.1 minutes in the multiple visits group. The average initial SBP and DBP were 148.3±20.0 mm Hg and 94.3±13.7 mm Hg, respectively, with a significant proportion of patients above guideline-based targets. In the study cohort, 62.4% of the patients (355) had an initial SBP reading greater than 140 mm Hg, and 80.5% (458) had an initial SBP reading greater than 130 mm Hg.

**Table:** Baseline Characteristics of the Patients^a^, ^b^

Baseline characteristic	(N=569)
Demographic features	
Age (y)	43.9±10.5
Sex	
Women	48.3 (275)
Men	51.7 (294)
Geographic location	
Rural	13.7 (78)
Urban/suburban	86.3 (491)
Comorbidities	
Overweight/obesity	12.0 (68)
Prediabetes	2.6 (15)
Diabetes	7.9 (45)
CKD	0.5 (3)
Dyslipidemia	15.5 (88)
Ischemic heart disease	0.5 (3)
COPD	1.5 (8)
Asthma	6.3 (36)
Initial SBP (mm Hg)	
<120	5.4 (31)
120-129	11.3 (64)
130-139	16.0 (91)
140-159	42.0 (239)
>160	25.3 (144)

a CKD, chronic kidney disease; COPD, chronic obstructive pulmonary disease.

b Categorical variables are presented as percentage (number). Continuous variables are presented as mean ± standard deviation.

**Figure 2:**
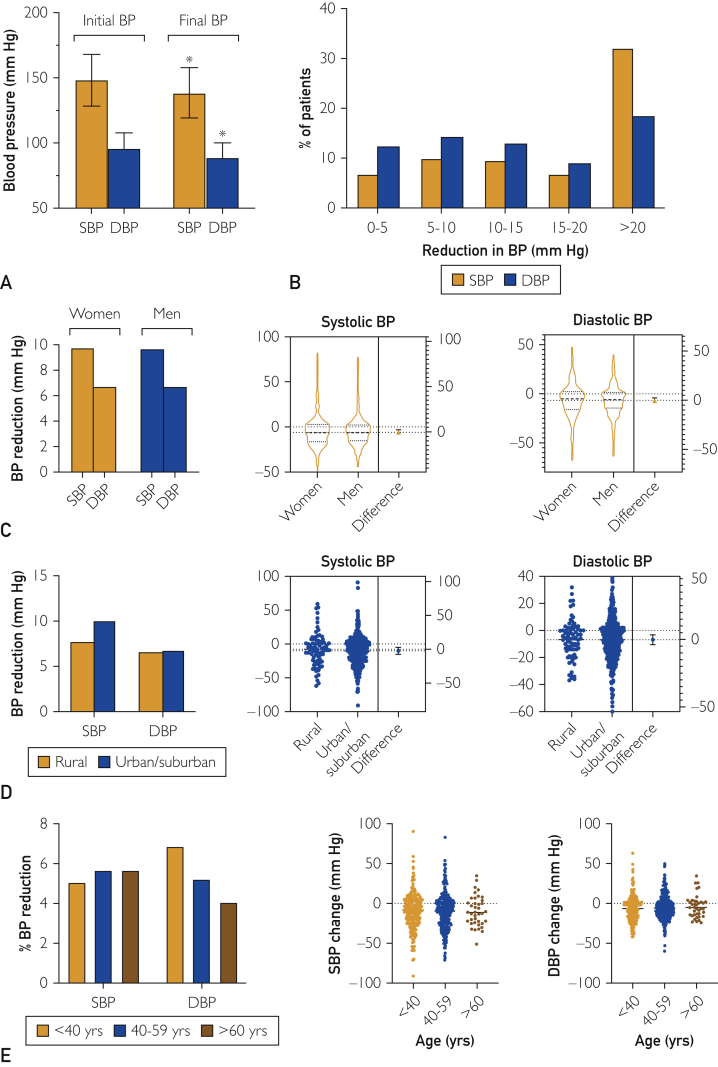
Effectiveness of a national video telemedicine platform in management of hypertension. A, Initial and final systolic blood pressure (SBP) and diastolic blood pressure (DBP) during the study period. B, Frequency distribution of blood pressure (BP) reduction across study patients. C, Blood pressure reduction stratified by sex. Left, SBP and DBP reductions stratified by sex; middle, estimation plot with difference of means for SBP; and right, estimation plot with difference of means of DBP. D, Blood pressure reduction stratified by geographic area (rural vs urban/suburban). Left, SBP and DBP reductions stratified by geographic region; middle, estimation plot with difference of means for SBP; and right, estimation plot with difference of means of DBP. E, Blood pressure reduction stratified by age (<40 years, 40 to 59 years, and >60 years). Left, SBP and DBP reductions stratified by age; middle, estimation plot with difference of means for SBP; and right, estimation plot with difference of means of DBP.

We examined the effectiveness of video telemedicine visits in the treatment of hypertension during the pandemic period by determining the reduction in recorded SBP and DBP across patients in the study cohort. When we compared initial and final recorded SBP and DBP during the study period, we found statistically significant reductions of 9.7 mm Hg and 6.8 mm Hg in SBP and DBP, respectively (*P*<.00001; Figure 2A). These changes corresponded to a 6.5% and 7.2% reduction in SBP and DBP, respectively. Most patients achieved a reduction of at least 5 mm Hg, with more than 48% of the patients achieving a reduction of at least 10 mm Hg in SBP (Figure 2B). We observed similar trends for DBP as well, with 54.0% of patients attaining a reduction of at least 5 mm Hg in DBP (Figure 2B). When we stratified patients across key demographic and clinical factors, we noted broad effectiveness of video visits with several important trends. We observed similar reductions in women and men for SBP (9.74 mm Hg vs 9.67 mm Hg; *P*=.97) and DBP (6.73 mm Hg vs 6.76 mm Hg; *P*=.98; Figure 2C). In addition, there were no significant differences in BP reduction by geographic location or the number of visits (Figure 2D). Patients residing in rural areas and urban/suburban areas had similar reductions in SBP (7.7 mm Hg vs 10.0 mm Hg; *P*=.38) and DBP (6.6 mm Hg vs 6.8 mm Hg; *P*=.92). Similarly, patients across different age ranges (<40 years, 40 to 59 years, >60 years) attained similar reductions in SBP (9.4 mm Hg vs 9.9 mm Hg vs 9.5 mm Hg; *P*=.95) and DBP (8.2 mm Hg vs 6.0 mm Hg vs 4.3 mm Hg; *P*=.16; Figure 2E). When we stratified patients by initial BP, we found that individuals who had higher baseline readings had a larger decrease in BP (data not shown). More than 70% of patients in the cohort (n=416) had an initial SBP above 140 mm Hg or DBP above 90 mm Hg. The mean reductions in SBP and DBP for these patients were −17.9 mm Hg and −12.8 mm Hg, respectively. In contrast, mean reductions in SBP and DBP for patients who started with an initial BP above 130/80 mm Hg were −14.2 mm Hg and −9.7 mm Hg, respectively.

**Figure 3:**
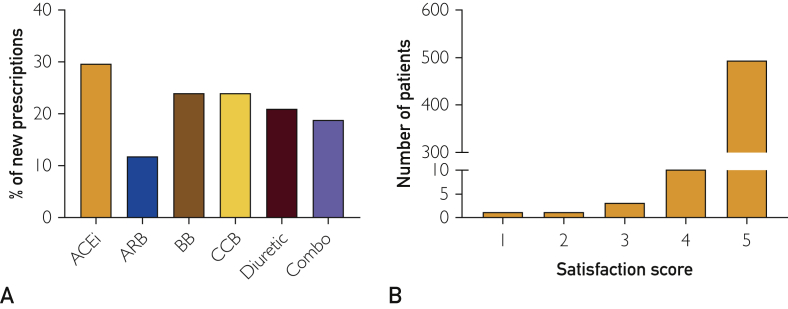
Prescription trends and patient satisfaction. A, Antihypertensive medications prescribed during the study period. B, Frequency of distribution for patient satisfaction survey scores. Scale of 1 to 5, with 5 representing most satisfied. ACEi, angiotensin-converting enzyme inhibitor; ARB, angiotensin receptor blocker; BB, beta blocker; CCB, calcium channel blocker.

When we examined prescriptions across the study period (Figure 3A), we found that 478 of the 569 patients were prescribed at least 1 new antihypertensive medication. The antihypertensives prescribed aligned well with ACC/AHA guideline recommendations. Angiotensin-converting enzyme inhibitors were prescribed most frequently (29.4%). A similar frequency of prescriptions was observed for diuretics (21.1%), calcium channel blockers (24.0%), and beta blockers (24.0%).

To evaluate patient satisfaction, we analyzed patient surveys that were automatically offered to each patient just after the end of each visit. Patients were asked to rate their overall satisfaction with the visit on a scale of 1 to 5, with 5 being the highest rating. Of the 569 patients analyzed, satisfaction survey data were available for 89.3% (n=508). The mean patient satisfaction score was 4.94/5.0, with 96.6% of the patients rating the visit either 4 or 5 (Figure 3). Clinicians receive these scores from all patients seen at the end of each workday along with optional typed feedback comments from each patient.

## Discussion

A rapidly growing number of reports in the literature describe the use of telemedicine video visits to develop systematic, large-scale efforts to promote hypertension treatment. A Cochrane review that analyzed outcomes of telehealth remote monitoring or video visits compared with in-person or telephonic visits for chronic illnesses such as congestive heart failure and diabetes found similar health outcomes.^8^ Reports on the use of telemedicine visits in Japan after the Great East Japan Earthquake of 2011 and in Europe during the middle months of the 2020 COVID-19 pandemic support the real-world effectiveness of these approaches for the management of a broad spectrum of conditions. However, several studies examining telemedicine in the United Kingdom, Scotland, and Italy before the pandemic reported varying levels of effectiveness of telehealth management of hypertension by primary care clinicians. Furthermore, an analysis of several million telemedicine visits in the United States during the 2020 pandemic found a 50% reduction in focused assessments of cardiovascular conditions such as hypertension compared with prior brick and mortar office visits for the same patients.^7^ As such, a 2020 AHA expert position paper that supports the use of telemedicine approaches to manage chronic conditions such as hypertension also highlights and underscores important concerns and limitations with purely telephonic telehealth visits.^9^

In this study, we conducted a retrospective analysis of telemedicine video visits in the management of hypertensive patients at home during the first year of the COVID-19 pandemic. As recommended by the most recent ACC/AHA guidelines for prevention, evaluation, and management of high BP in adults, the national virtual practice in this study employed a holistic approach with attention to nonpharmacologic lifestyle interventions, such as the Dietary Approaches to Stop Hypertension diet, increasing exercise, controlling weight, limiting alcohol consumption, and stopping smoking.^9^ Physicians and providers in this national virtual medical practice received extensive training in telemedicine webside manner and communication skills. Most of the physicians also received training by either direct or indirect participation in our hypertension clinical education module emphasizing the ACC/AHA guidelines as a part of Doctor on Demand’s internal continuing medical education program or as part of recent board recertification.

Our data showed strong patient engagement and broad effectiveness of the video telemedicine platform in managing hypertension. Patients experienced significant decreases in SBP and DBP with video telemedicine visits during the pandemic period. We found that patients who had higher initial BP readings realized the greatest reductions in BP. Otherwise, we noted similar reductions in SBP and DBP across women and men, different age ranges, and geographic locations (rural vs urban/suburban locations). These results were achieved over an average of 3 visits per patient during the study period of 1 year, with each visit lasting just more than 12 minutes on average. We also evaluated associated measures of patient satisfaction with these visits and noted high scores. Collectively, these results suggest that video telemedicine platforms offer an efficient, effective, and patient-centered approach to address important gaps in hypertension management, including access to care and patient engagement.

Most published literature on telemedicine approaches to management of hypertension have not focused on synchronous video visits by specifically trained virtualist physicians and nurse practitioners, which is 1 of the key characteristics of this report. The findings can arguably represent general effectiveness of telemedicine video visits by a trained, predominantly physician workforce in achieving hypertension control by generally accepted guidelines and by attention to a holistic, patient-centered approach within a few visits. Notably, the quality of these visits was highly acceptable to this national cohort of 569 patients, who returned for video visits an average of 3.1 visits during the study year. We also note that BP was documented an average of 6.4 times over the average of 3.1 visits, suggesting that the physicians and patients generally were attempting to follow ACC/AHA guidelines. We note that these results serve as a baseline for “usual care” by telemedicine video visits. Future work on team-based care by telemedicine video visits may strive to improve on these baseline results, with a patient-centered medical home approach,^10^ especially looking toward the percentage of patients in the population achieving an agreed BP control goal, as spotlighted by the AMA Target: BP program and the Centers for Disease Control and Prevention Million Hearts program. These results are similar to results for usual care reported in other hypertension population health studies by top-performing health systems, such as Kaiser-Permanente Northern California, in recent years.^11^ Patient satisfaction with telemedicine visits has been reported in several studies.^12^, ^13^, ^14^ Patient satisfaction specifically with hypertension care by video visits is reflected in this study, which we believe to be the first report of such findings.

Our study has a number of limitations, including the self-measurement of patient-reported BP, in addition to variability in the home devices used, which could not be standardized. In addition, our database does not include information on care received outside of the virtual care practice network studied in this investigation. Our data are on a commercially insured population, and comprehensive assessment of all patient comorbidities and ethnic/racial identifications are not captured in our database. Also, these results may not be generalizable to clinician workforces who have not been trained in the evaluation and management of patients by a telemedicine video visit. This study is not systematically comparing home video visits with in-office visits. This is an observational study, and the analysis is not comparing an intervention with a control group in a randomized clinical trial. We are aware that some experts recommend obtaining twice-daily SBP readings for ideally 5 to 7 consecutive days.^3^ Following such a strict protocol is not practical in terms of patient expectations and satisfaction with real-world telemedicine visits.

## Conclusion

Virtualist visits for hypertension in the United States during the pandemic were generally effective when access to primary physician care was disrupted. The holistic approach plus ACC/AHA guideline-adherent care may be key. Reductions in SBP and DBP compare favorably with in-person settings. Patient satisfaction scores were high. These results are a baseline for future population-level hypertension and cardiovascular risk interventions for populations with suboptimal access to quality health care across many rural and urban zip codes. Readers should be reminded that this study is focused on a national telemedicine practice that is providing access to quality care in a national health emergency, in conditions that are suboptimal for running detailed research protocols. We should also be reminded that follow-up by patients was good, during several visits in this cohort, which also speaks to the practical utility of these visits and patient acceptance of and adherence to the holistic advice to achieve hypertension control.

The results of this study suggest that a holistic approach to hypertension management, attending to lifestyle changes and appropriate medications, is effective through virtual video primary care visits nationwide. We did not find notable differences between patients in terms of rural or metro location, sex, ethnicity, or other factors, other than adherence to therapy. The few patients whose BP did not improve were patients who admitted they were nonadherent to lifestyle or medication recommendations. Additional research to understand the barriers to care for this subset of the population of telemedicine patients may yield useful insights. Patient satisfaction with these virtual hypertension visits was high. These findings further support our colleagues’ previous findings that clinician-patient relationships are established and well maintained in a virtual video visit model.^6^

## References

[1] Whelton P.K., Carey R.M., Aronow W.S. (2018). 2017 ACC/AHA/AAPA/ABC/ACPM/AGS/APhA/ASH/ASPC/NMA/PCNA Guideline for the Prevention, Detection, Evaluation, and Management of High Blood Pressure in Adults: a report of the American College of Cardiology/American Heart Association Task Force on Clinical Practice Guidelines [erratum appears in Hypertension. 2018;71(6):e140-e144]. Hypertension.

[2] Curfman G., Bauchner H., Greenland P. (2020). Treatment and control of hypertension in 2020: the need for substantial improvement. JAMA.

[3] Muntner P., Hardy S.T., Fine L.J. (2020). Trends in blood pressure control among US adults with hypertension, 1999-2000 to 2017-2018. JAMA.

[4] Abdulla J., Barlera S., Latini R. (2007). A systematic review: effect of angiotensin converting enzyme inhibition on left ventricular volumes and ejection fraction in patients with a myocardial infarction and in patients with left ventricular dysfunction. Eur J Heart Fail.

[5] Kendzerska T., Zhu D.T., Gershon A.S. (2021). The effects of the health system response to the COVID-19 pandemic on chronic disease management: a narrative review. Risk Manag Healthc Policy.

[6] Uscher-Pines L., Thompson J., Taylor P. (2020). Where virtual care was already a reality: experiences of a nationwide telehealth service provider during the COVID-19 pandemic. J Med Internet Res.

[7] Alexander G.C., Tajanlangit M., Heyward J., Mansour O., Qato D.M., Stafford R.S. (2020). Use and content of primary care office–based vs telemedicine care visits during the COVID-19 pandemic in the US. JAMA Netw Open.

[8] Flodgren G., Rachas A., Farmer A.J., Inzitari M., Shepperd S. (2015). Interactive telemedicine: effects on professional practice and health care outcomes. Cochrane Database Syst Rev.

[9] Omboni S., McManus R.J., Bosworth H.B. (2020). Evidence and recommendations on the use of telemedicine for the management of arterial hypertension: an international expert position paper. Hypertension.

[10] Parameswaran V., Josan K., Winterbottom J. (2019). CardioClick an innovative telehealth approach to lifestyle intervention in high risk South Asians. Circulation.

[11] Jaffe M.G., Lee G.A., Young J.D., Sidney S., Go A.S. (2013). Improved blood pressure control associated with a large-scale hypertension program. JAMA.

[12] Polinski J.M., Barker T., Gagliano N. (2016). Patients' satisfaction with and preference for telehealth visits. J Gen Intern Med.

[13] Martinez K.A., Rood M., Jhangiani N. (2018). Patterns of use and correlates of patient satisfaction with a large nationwide direct to consumer telemedicine service. J Gen Intern Med.

[14] Elliott T., Tong I., Sheridan A., Lown B.A. (2020). Beyond convenience: patients' perceptions of physician interactional skills and compassion via telemedicine. Mayo Clin Proc Innov Qual Outcomes.

